# Circulating CXCL9, monocyte percentage, albumin, and C-reactive protein as a potential, non-invasive, molecular signature of carotid artery disease in 65+ patients with multimorbidity: a pilot study in Age.It

**DOI:** 10.3389/fendo.2024.1407396

**Published:** 2024-07-23

**Authors:** Miriam Capri, Sara Fronterrè, Salvatore Collura, Enrico Giampieri, Sara Carrino, Francesca Maria Feroldi, Erika Ciurca, Maria Conte, Fabiola Olivieri, Ines Ullo, Rodolfo Pini, Andrea Vacirca, Annalisa Astolfi, Francesco Vasuri, Gaetano La Manna, Gianandrea Pasquinelli, Mauro Gargiulo

**Affiliations:** ^1^ Department of Medical and Surgical Sciences (DIMEC), Alma Mater Studiorum-University of Bologna, Bologna, Italy; ^2^ Interdepartmental Centre - Alma Mater Research Institute on Global Challenges and Climate Change, University of Bologna, Bologna, Italy; ^3^ Vascular Surgery Unit, IRCCS Azienda Ospedaliero-Universitaria di Bologna, Bologna, Italy; ^4^ Department of Clinical and Molecular Sciences, Università Politecnica delle Marche, Ancona, Italy; ^5^ Clinic of Laboratory and Precision Medicine, IRCCS INRCA, Ancona, Italy; ^6^ Nephrology, Dialysis and Renal Transplant Unit, IRCCS Azienda Ospedaliero-Universitaria di Bologna, Bologna, Italy; ^7^ IRCCS Azienda Ospedaliero-Universitaria di Bologna, Bologna, Italy; ^8^ Pathology Unit, IRCCS Azienda Ospedaliero-Universitaria di Bologna, Bologna, Italy

**Keywords:** microRNAs, carotid artery disease, biomarkers, asymptomatic patients, symptomatic patients, elders, CXCL9, IL-6

## Abstract

**Background:**

Carotid endarterectomy (CEA) for the prevention of upcoming vascular and cerebral events is necessary in patients with high-grade stenosis (≥70%). In the framework of the Italian National project Age.It, a pilot study was proposed aiming at the discovery of a molecular signature with predictive potential of carotid stenosis comparing 65+ asymptomatic and symptomatic inpatients.

**Methods:**

A total of 42 inpatients have been enrolled, including 26 men and 16 women, with a mean age of 74 ± 6 years. Sixteen symptomatic and 26 asymptomatic inpatients with ≥70% carotid stenosis underwent CEA, according to the recommendations of the European Society for Vascular Surgery and the Society for Vascular Surgeons. Plaque biopsies and peripheral blood samples from the same individuals were obtained. Hematobiochemical analyses were conducted on all inpatients, and plasma cytokines/molecules, such as microRNAs (miRs), IL-6, sIL-6Ralpha, sgp130, myostatin (GDF8), follistatin, activin A, CXCL9, FGF21, and fibronectin, were measured using the ELISA standard technique. MiR profiles were obtained in the discovery phase including four symptomatic and four asymptomatic inpatients (both plasma and plaque samples), testing 734 miRs. MiRs emerging from the profiling comparison were validated through RT-qPCR analysis in the total cohort.

**Results and conclusion:**

The two groups of inpatients differ in the expression levels of blood c-miRs-126–5p and -1271–5p (but not in their plaques), which are more expressed in symptomatic subjects. Three cytokines were significant between the two groups: IL-6, GDF8, and CXCL9. Using receiver operating characteristic (ROC) analysis with a machine learning-based approach, the most significant blood molecular signature encompasses albumin, C-reactive protein (CRP), the percentage of monocytes, and CXCL9, allowing for the distinction of the two groups (AUC = 0.83, 95% c.i. [0.85, 0.81], *p* = 0.0028). The potential of the molecular signature will be tested in a second cohort of monitored patients, allowing the application of a predictive model and the final evaluation of cost/benefit for an assessable screening test.

## 1 Introduction

Epidemiological studies reveal that 21% of individuals aged 30–79 years have carotid plaque, defined as a focal carotid intima-media thickness of 0.5–1.5 mm or more invading into the lumen (≤50 of stenosis), and 1.5% of individuals have carotid stenosis (≥50%), equivalent to approximately 816 million people with carotid plaque and 58 million with carotid stenosis (1). As is well recognized, people with carotid plaque or carotid stenosis are at an increased risk of developing systemic cardiovascular diseases (CVDs) (2–4), and carotid endarterectomy (CEA) is deemed necessary in patients with high-grade stenosis (≥70%) to prevent forthcoming vascular and cerebral events.

In this perspective, the possibility to monitor the development and the stability of carotid plaque is clinically relevant especially for asymptomatic and elderly patients. On the other hand, symptomatic patients are those subjects who have manifested signs of prior cerebrovascular events, such as transient ischemic attack, ischemic stroke, and transient monocular vision loss, and urgently require surgical intervention of carotid artery (stenosis ≥70%). Concurrently, the identification of asymptomatic patients with carotid plaque has been recommended in assessing cardiovascular risk (5), and various circulating blood biomarkers may aid in the faster identification of carotid atherosclerosis. This research area has been growing during last decade, focusing on immune system cell sub-populations, pro-inflammatory molecules, and tissue/blood circulating microRNAs (c-miRs) (6).

Our team has recently investigated the differences in miR profiling expression within the human artery wall, revealing miR-related differences in the carotid, femoral, and abdominal aortic arteries in both health and pathological conditions (atheroma) (7, 8), and other researchers have deeply investigated plaque stability/instability and associated miR expression (9–11). In the light of these previous results, the primary object of the current work has been to identify a miR-based molecular signature to distinguish asymptomatic and symptomatic older (≥65 years) inpatients in blood and plaques, plausibly.

The use of c-miRs and cytokines/chemokines as potential biomarkers has been well recognized for both the aging process and different pathologies/age-related diseases (6, 12–17). C-miRs are protected from plasma/serum RNase activity through their transport on RNA–protein complexes with lipoproteins or Ago2 proteins (18, 19), as well as through their inclusion inside extracellular nano-microvesicles. In the current work, c-miRs have been obtained by plasma RNA extraction including all the components and assessed by standard RT-qPCR. Furthermore, the selection of specific cytokines/chemokines, in accordance with the ongoing Italian National project (PNRR-PE8: Age.It at the website: https://ageit.eu/wp/), has been proposed for the implementation of the molecular signature. Cytokines/chemokines, including IL-6, CCL2, CXCL9, and CXCL12, are recognized to be important drivers of atheroma development and the inflammatory microenvironment (20–24).

The first hypothesis testing aimed to identify a common miR-based signature between blood c-miRs and carotid atheroma, likely able to distinguish between asymptomatic and symptomatic patients. The second hypothesis testing focused on specific cytokines/molecules, such as IL-6, sIL-6Ralpha, sgp130, GDF8, follistatin, activin A, CXCL9, FGF21, and fibronectin. The objective was to enhance the potential miR-based signature, with the aim of better distinguishing between asymptomatic and symptomatic patients.

As part of the Italian National project (Age.It), the envisioned final achievement will be the validation of the identified molecular signature in patients with different carotid stenosis degree during the monitoring period and the possibility of applying a predictive model.

## 2 Materials and methods

### 2.1 Pilot study and patients’ recruitment

The current pilot study focused on 42 individuals, comprising 26 asymptomatic (11 women and 15 men) and 16 symptomatic (5 women and 11 men) inpatients diagnosed with carotid artery disease. The two groups had a mean age of 72 ± 5 years and 77 ± 7 years, respectively. Recruitment was carried out after obtaining approval from the regional ethical committee (88/2019/Sper/AOUBo) and following an amendment (n. EM511–2023) for the project inclusion in Age.it (PNRR-PE8).

All recruited inpatients showed carotid artery stenosis ≥70% and underwent CEA according to the recommendations of the European Society for Vascular Surgery and the Society for Vascular Surgeons, since 70% of stenosis is a critical threshold for urgent surgical intervention. Symptomatic carotid stenosis was defined as the occurrence of ipsilateral cerebral ischemic events (major or minor stroke, TIA, or amaurosis fugax) within the last 6 months. CEA-derived biopsies (plaques) and blood samples (before CEA) were collected from each inpatient. Neurological symptoms (amaurosis fugax, TIA, and minor and major stroke), vascular risk factors, morbidities or comorbidities (hypertension, coronary artery disease, chronic obstructive pulmonary disease, dyslipidemia, type II diabetes, current smoking, and chronic renal failure), and current therapies were recorded. Exclusion criteria were current acute illnesses; hepatic, severe renal, or cardiac insufficiency; obesity; and type II diabetes with severe complications.

All inpatients were also tested for hemato-biochemical parameters, i.e., white blood cell count (WBC), erythrocytes (RBC), hemoglobin (HGB), hematocrit (HCT), mean corpuscular volume (MCV), the average hemoglobin content in erythrocytes (MCH), mean hemoglobin concentration in erythrocytes (MCHC), red blood cell distribution (% RDW-CV, RDW-SD), neutrophils, lymphocytes, monocytes, eosinophils, basophils, platelets, mean platelet volume (MPV), urea, creatinine, estimated glomerular filtration rate (eGFR), uric acid, sodium, potassium, total calcium, total bilirubin, direct bilirubin, indirect bilirubin, aspartate aminotransferase (AST), alanine aminotransferase (ALT), γ-glutamyl transferase (GGT), total amylase, creatine kinase (CK), lactate dehydrogenase (LDH), C-reactive protein, albumin, total proteins, glucose, total cholesterol, high-density lipoprotein (HDL) cholesterol, LDL (low-density lipoprotein) cholesterol, and triglycerides (TGC).

### 2.2 Sample processing

Whole blood was collected from 42 inpatients before surgery in vacuum tubes following standard procedures and processed within 1 h. The tubes were centrifuged at 2,500 × *g* for 20 min at 4°C for plasma separation and aliquoted in cryotubes for long-term storage at −80°C. Carotid plaques were completely removed during surgery to preserve the plaque structure. After 24 h of decalcification, samples were cut into serial sections and the area with the highest percentage of stenosis was identified and defined for the analysis. Biopsies from 41 inpatients were formalin-fixed paraffin-embedded (FFPE) and routinely processed. Carotid atherosclerotic lesions were defined according to the American Heart Association classification and grouped as hemorrhagic or non-hemorrhagic plaque.

### 2.3 RNA extraction from plasma

Total RNA was extracted from plasma samples using the Total RNA Purification Kit (Norgen Biotek Corporation, Thorold, Ontario, Canada) and strictly adhering to the manufacturer’s instructions. Additionally, cel-miR-39 (Norgen) was spiked into each sample as an internal control for RNA extraction in plasma samples. On average, 100 μL of plasma produced 18 ± 5 ng/μL total RNA. The extracted RNA was quantified using Nanodrop^TM^ One/OneC Microvolume UV-Vis 1000 spectrophotometer (Thermo Fisher Scientific, Waltham, MA, USA).

### 2.4 RNA extraction from formalin-fixed, paraffin-embedded tissue

Total RNA was extracted from formalin-fixed paraffin-embedded (FFPE) sections. Each slice of tissue (20 µm thick) produced 19 ± 7 ng/µL total RNA on average. Briefly, four slices from each biopsy block were deparaffinized (∼20 min) and digested with protease (overnight at 50°C and 15 min at 80°C). RNA extraction was obtained using a commercial kit (RecoverAll™ Total Nucleic Acid Isolation Kit for FFPE, Thermo Fisher Scientific, Waltham, MA, USA), which allows isolation of RNA including miRs, following the manufacturer’s instructions. Subsequently, the extracted RNA was quantified using a Nanodrop^TM^ One/OneC Microvolume UV-Vis spectrophotometer (Thermo Fisher Scientific, Waltham, MA, USA).

### 2.5 Discovery phase with miR profiling

MiR profiles were obtained from eight selected inpatients, i.e., four symptomatic (two M, two F; age 75 ± 9 years; max stenosis of 80%) and four asymptomatic (two M, two F; age 76 ± 4 years; max stenosis of 95%), using human miR microfluidic cards for the assessment of 754 miRs (TaqMan Array Human MicroRNA A+B Cards/arrays; Applied Biosystems by Thermo Fisher Scientific, Waltham, MA, USA).

The inpatients were chosen for advanced age and maximum degree of occlusion in both carotid arteries. Carotid plaque specimens and plasma samples from the same patient were analyzed.

Six microliters of total RNA at the concentration of 4.2 ng/µL were used for the card/array analysis of the biopsies, and similarly, 6 µL of total RNA obtained from plasma samples was used for the card/array analysis. RNA was converted to cDNA by priming with a mixture of looped primers and then pre-amplified using the MegaPlex™ primer pools (Applied Biosystems by Thermo Fisher Scientific, Waltham, MA, USA) according to the manufacturer’s instructions, as previously published (25). The assay was performed using Applied Biosystems 7900 HT real-time PCR instrument. All the arrays/cards were normalized using the median of the overall miR expression on each array, thus obtaining ΔCt values. Only miRs expressed in all samples were selected for analyses and Ct values ≤ 30 were set as cutoff. To compare symptomatic and asymptomatic groups, the fold change (FC) were calculated. It was calculated on the estimated mean difference of ΔCt values between the two groups as 2^−ΔΔCt^, as applied in previous works (25). FC ≥ 2 and ≤ −2 were selected, thus obtaining miR profiling for further validation.

### 2.6 Validation phase with RT-qPCR in both plasma and plaques

RT-qPCR assays were performed on selected miRs that showed variation in FC values (FC ≥ 2 or ≤ −2) comparing symptomatic and asymptomatic inpatients in both plaque and plasma samples, considering a plausible cross-talk between blood circulating molecules and carotid artery wall tissue.

The analysis was extended to all subjects using TaqMan technologies (Thermo Fisher Scientific, Waltham, MA, USA) following the manufacturer’s protocol. Five microliters of extracted RNA (both from plaque and plasma) were transcribed to cDNA using the TaqMan MicroRNA Reverse Transcription Kit (Applied Biosystems, by Thermo Fisher Scientific, Waltham, MA, USA), followed by RT-qPCR with TaqMan MicroRNA Assays (Thermo Fisher Scientific cod. 4427975). The following assays were used: hsa-miR-134–5p (ID cod. 000459), hsa-miR-145–5p (ID cod. 002278), hsa-miR-151a-5p (ID cod. 002642), hsa-miR-34b-3p (ID cod. 002102), hsa-miR-451 (ID cod. 001105), hsa-miR-720 (ID cod. 002895), hsa-miR-126–5p (ID cod. 000451), and hsa-miR-1271–5p (ID cod. 002779). Data normalization was performed using 20 fmol of cel-mir-39 (Qiagen, Hilden, Germany) for plasma samples while hsa-miR-16 (ID cod. 000391) was adopted for biopsies among other housekeepers tested. The relative expression of all miRs was determined using the standard formula: 2^−ΔCt^.

### 2.7 Plasma cytokine/molecule assessment

All the cytokines/molecules, i.e., IL-6, sIL-6Ralpha, sgp130, GDF8, follistatin, activin A, CXCL9, FGF21, and fibronectin, were assessed with the Human Quantikine ELISA Kit in plasma samples using commercial kits (R&D, Minneapolis, USA) with catalog numbers D6050, DR600, DGP00, DGDF80, DFN00, DAC00B, DCX900, DF2100, and DFBN10, respectively. All samples were analyzed in duplicate according to the manufacturer’s instructions.

### 2.8 Statistical analysis

Statistical analysis was performed using both the software SPSS v.26 and Python v 3.11.7/scikit-learn v 1.3.2. Statistical significance was defined as *p*-value ≤ 0.05; Student’s *t*-test for unpaired and parametric data was applied to compare variables between symptomatic and asymptomatic groups.

The model for patient classification and molecular signature identification was developed from the full dataset (hemato-biochemical analysis, cytokines, and miRs). To ensure the model robustness, all analyses were performed using a leave-one-out cross-validation approach, where the model was fitted repeatedly on a subset of all the patients except for one, and the prediction was assessed on the left out one, repeating this procedure to obtain a prediction for every subject. Missing data were estimated using an Iterative Imputation algorithm (26) only on the training subset, then each feature was standardized to mean 0 and standard deviation 1. The most important features were selected using a Sequential Feature Selection method (27), based on the usefulness of the features for a Random Forest Classifier, starting with no features and adding them progressively based on the obtained improvement in a secondary leave-one-out cross-validation. The actual prediction was performed training a Random Forest Classifier with 100 decision trees. This prediction method allows obtaining both a binary prediction (symptomatic or asymptomatic) and a continuous one, with a degree of similarity to each class (going from 0, totally different, to 1, completely similar). The quality of the prediction for each set of features was estimated using the Area Under the Receiver Operating Curve (AUC-ROC) with 10 replications for each model, thus obtaining an average AUC value ± SD with the related confidence interval for each model, to account for the randomized initialization of the components of the whole prediction pipeline (imputation, sequential feature selection, and Random Forest Classifier).

## 3 Results

### 3.1 Pilot study

The two cohorts of the 26 asymptomatic and 16 symptomatic inpatients were assessed for hemato-biochemical parameters (**Table 1**). By comparing the two groups, the percentage of monocytes and albumin were significantly different (*p* = 0.036 and *p* = 0.005, respectively).

**Table 1:** Asymptomatic and symptomatic recruited inpatients and hemato-biochemical analysis (Student’s *t*-test).

Parameters	N°	Asymptomatic Mean ± SD	Symptomatic Mean ± SD	*p*
Age	42	72 ± 5	77 ± 7	
RBC (×10^6^/μL)	42	4.4 ± 0.5	4.2 ± 0.6	n.s.
WBC (×10^3^/μL)	42	8 ± 2	7 ± 2	n.s.
HGB (g/dL)	42	13.4 ± 1.2	12.8 ± 1.9	n.s.
MCV (fL)	42	89 ± 6	90 ± 5	n.s.
MCH (pg)	42	31 ± 2	30 ± 2	n.s.
MCHC (g/dL)	42	34 ± 1	34 ± 1	n.s.
RDW-CV (%)	41	13.2 ± 0.9	13.5 ± 0.9	n.s.
RDW-SD (fL)	41	42.8 ± 3.9	43.9 ± 4.2	n.s.
Neutrophils (%)	41	65 ± 7	64 ± 8	n.s.
Lymphocytes (%)	41	26 ± 6	26 ± 8	n.s.
Monocytes (%)	41	6.5 ± 1.5	7.6 ± 1.6	**0.036**
Eosinophils (%)	41	2.2 ± 1.3	2.0 ± 1.7	n.s.
Basophils (%)	41	0.4 ± 0.2	0.5 ± 0.3	n.s.
Platelets (×10^3^/μL)	42	229 ± 50	229 ± 69	n.s.
Neutrophils/Lymphocytes	41	2.7 ± 0.8	2.8 ± 1.2	n.s.
MPV (fL)	42	11 ± 1	11 ± 1	n.s.
Urea (mg/dL)	42	39 ± 12	36 ± 11	n.s.
Creatinine (mg/dL)	42	1.06 ± 0.31	1.04 ± 0.27	n.s.
eGFR (mL/min)	42	80 ± 26	79 ± 20	n.s.
Uric acid (mg/dL)	42	5.42 ± 1.13	5.31 ± 1.65	n.s.
Sodium (mmol/L)	42	140 ± 2	141 ± 3	n.s.
Calcium tot (mg/dL)	42	9.37 ± 0.49	9.53 ± 0.52	n.s.
Total proteins (g/dL)	42	6.67 ± 0.41	6.62 ± 0.46	n.s.
Albumin (g/dL)	31	4.1 ± 0.2	3.8 ± 0.4	**0.005**
Total bilirubin (mg/dL)	42	0.65 ± 0.31	0.76 ± 0.36	n.s.
Direct bilirubin (mg/dL)	42	0.22 ± 0.09	0.27 ± 0.12	n.s.
Indirect bilirubin (mg/dL)	42	0.43 ± 0.22	0.49 ± 0.25	n.s.
AST (GOT) (U/L)	42	24 ± 16	24 ± 8	n.s.
ALT (GPT) (U/L)	42	22 ± 18	23 ± 15	n.s.
GGT (U/L)	42	26 ± 14	54 ± 77	n.s.
Total amylase (U/L)	42	76 ± 29	84 ± 27	n.s.
Creatine Kinase (U/L)	42	139 ± 144	100 ± 87	n.s.
Glucose (mg/mL)	42	112 ± 34	96 ± 10	n.s.
Total cholesterol (mg/dL)	42	150 ± 26	149 ± 46	n.s.
LDL (mg/dL)	42	81 ± 23	83 ± 36	n.s.
HDL (mg/dL)	42	48 ± 9	47 ± 17	n.s.
Triglycerides (mg/dL)	42	131 ± 40	119 ± 47	n.s.
Lactate dehydrogenase (U/L)	42	189 ± 52	179 ± 46	n.s.
C-reactive protein (mg/dL)	37	0.46 ± 0.76	1.08 ± 1.96	n.s.

In bold the significant p values. (< 0.05). ns, not significant.

### 3.2 Carotid plaques between asymptomatic and symptomatic

Histological analysis showed the presence of an atheromatous plaque in 41 obtained biopsies with minimal differences in the histological features of the atherosclerotic specimens. For this reason, attention was focused on the presence or absence of intraplaque hemorrhage as evidence of plaque vulnerability and susceptibility to rupture, potentially associated with the clinical setting of symptomatic/asymptomatic nature.

Carotid plaque specimens were therefore grouped as hemorrhagic plaque (*N* = 20) or non-hemorrhagic plaque (*N* = 21). The hemorrhagic plaques included 10 specimens from asymptomatic inpatients and 10 specimens from symptomatic inpatients. Conversely, the group of non-hemorrhagic plaques consisted of 15 specimens from asymptomatic and 6 specimens from symptomatic inpatients. Comparing the hemorrhagic with the non-hemorrhagic group, no significant differences in the selected, plaque-derived miRs and c-miRs expression levels were observed (data not shown).

### 3.3 MiRs profiling in carotid plaque and plasma from the discovery phase

**Figure 1:**
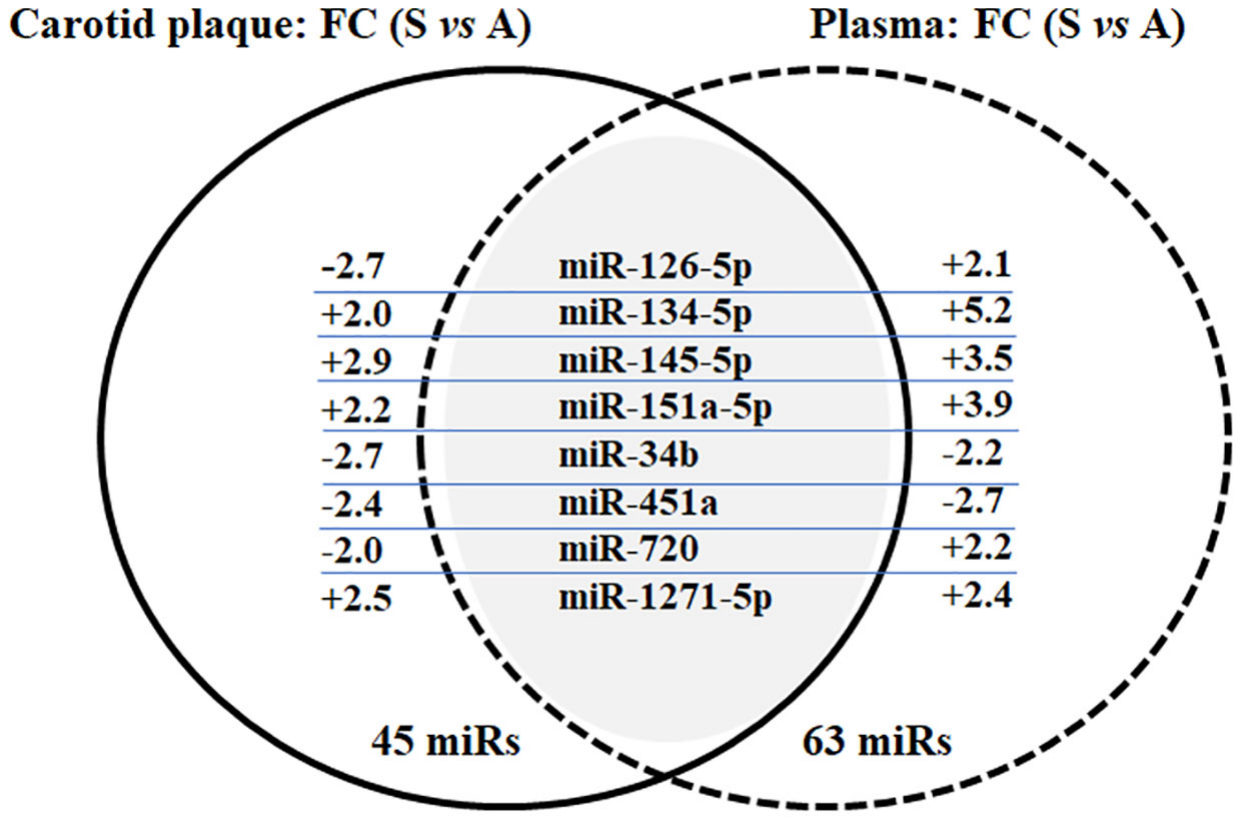
Venn diagram of common miRs between carotid plaque and plasma in the selected asymptomatic and symptomatic inpatients. Results of miR profiling in terms of shared miRs and FC (comparing asymptomatic and symptomatic inpatients, FC ≥ 2 and FC ≤ −2) in both carotid plaque and plasma. Data were validated in single RT-qPCR; only miR-126–5p and miR-1271–5p have been confirmed in plasma samples (see **Supplementary Table S3**; **Table 2**).

MiRs profiling was performed to identify miR changes in plasma and carotid plaque from the same individual, comparing extreme phenotypes of symptomatic and asymptomatic inpatients. The average of detected miRs with card A was 205 in plasma samples and 279 in biopsies (miRs with Ct < 32 were 159 and 250, respectively), while the average detected with card B was 124 in plasma samples and 188 in biopsies (miRs with Ct < 32 were 71 and 147, respectively). Putative differentially expressed miRs were identified according to FC values (FC ≥ 2 and FC ≤ −2). A total of 45 and 63 miRs were found to satisfy such prerequisites in biopsy and plasma, respectively. All the data related to profiling and comparison of asymptomatic versus symptomatic inpatients are included as **Supplementary Material** (**Supplementary Tables S1**, **S2**) for both atheroma biopsies and plasma. When putative differently expressed miRs were compared between carotid plaque and plasma, eight shared miRs were identified: miR-126–5p, miR-134–5p, miR-145–5p, miR-151a-5p, miR-34b, miR-451a, miR-720, and miR-1271–5p (**Figure 1**). The figure also shows the FC between symptomatic and asymptomatic groups, while the relative expression average ( ± SD) is reported in **Supplementary Table S3**.

### 3.4 MiRs validation in carotid plaque and plasma

Validation through RT-qPCR analysis was performed for the selected miRs in the total cohort of subjects, i.e., 26 asymptomatic and 16 symptomatic inpatients. Carotid plaques of all inpatients were tested by RT-qPCR for single miR analysis of miR-126–5p, miR-1271–5p, miR-145–5p, miR-151a-5p, miR-451a, and miR-720, but results did not confirm data obtained by the miR profiling, and no significant differences were observed (**Table 2**). Plasma samples were also tested by RT-qPCR for single miR analysis of the above mentioned miRs comparing asymptomatic and symptomatic groups, and borderline and/or significant difference were reported for miR-126–5p (*p* = 0.0574) and miR-1271–5p (*p* = 0.0396), being more expressed in symptomatic inpatients (**Table 2**), in accordance with the trend observed through card/array technology. Conversely, miR-145–5p, miR-151a-5p, miR-451a, and miR-720 showed no significant difference in expression levels between asymptomatic and symptomatic inpatients (**Table 2**).

**Table 2:** Relative expression of the selected miRs obtained through RT-qPCR both in plaque biopsies and in plasma from the same inpatients.

miRs (RT-qPCR)	Plaque-A Mean ± SD	Plaque-S Mean ± SD	A - S (*n*) - (*n*)	p	Plasma-A Mean ± SD	Plasma-S Mean ± SD	A - S (*n*) - (*n*)	*p*
miR-126–5p	0.00953 ± 0.00291	0.01061 ± 0.00561	25 - 16	ns	0.00523 ± 0.00367	0.00776 ± 0.00468	26 - 16	**0.0056**
miR-145–5p	3.0 ± 1.5	3.0 ± 2.0	25 - 16	ns	0.00057 ± 0.00058	0.00093 ± 0.00075	26 - 16	ns
miR-151–5p	0.01634 ± 0.00572	0.01809 ± 0.00738	25 - 16	ns	0.00144 ± 0.00139	0.00227 ± 0.00192	26 - 16	ns
miR-451a	0.34360 ± 0.30602	0.39386 ± 0.21340	25 - 16	ns	0.42864 ± 0.24105	0.41205 ± 0.28108	26 - 16	ns
miR-720	29.11 ± 17.86	30.51 ± 13.87	25 - 16	ns	0.00213 ± 0.00113	0.00265 ± 0.00151	26 - 16	ns
miR-1271–5p	0.00118 ± 0.00031	0.00116 ± 0.00027	25 - 16	ns	0.000039 ± 0.000018	0.000057 ± 0.000037	26 - 16	**0.0396**
miR-134	0.00088 ± 0.00040	0.00063 ± 0.00042	22 - 14	ns	0.00048 ± 0.00036	0.00053 ± 0.00035	26 - 14	ns
miR-34b	0.00063 ± 0.00035	0.00049 ± 0.00036	20 - 12	ns	0.00038 ± 0.00039	0.00057 ± 0.00037	23 - 12	ns

A, Asymptomatic; S, Symptomatic inpatients. Some assays had Ct > 32 and they were excluded, as shown. In bold the significant p values. (≤ 0.05). ns, not significant. Statistical analysis was performed with Student’s t-test.

### 3.5 Plasma cytokines and the molecular signature

**Figure 2:**
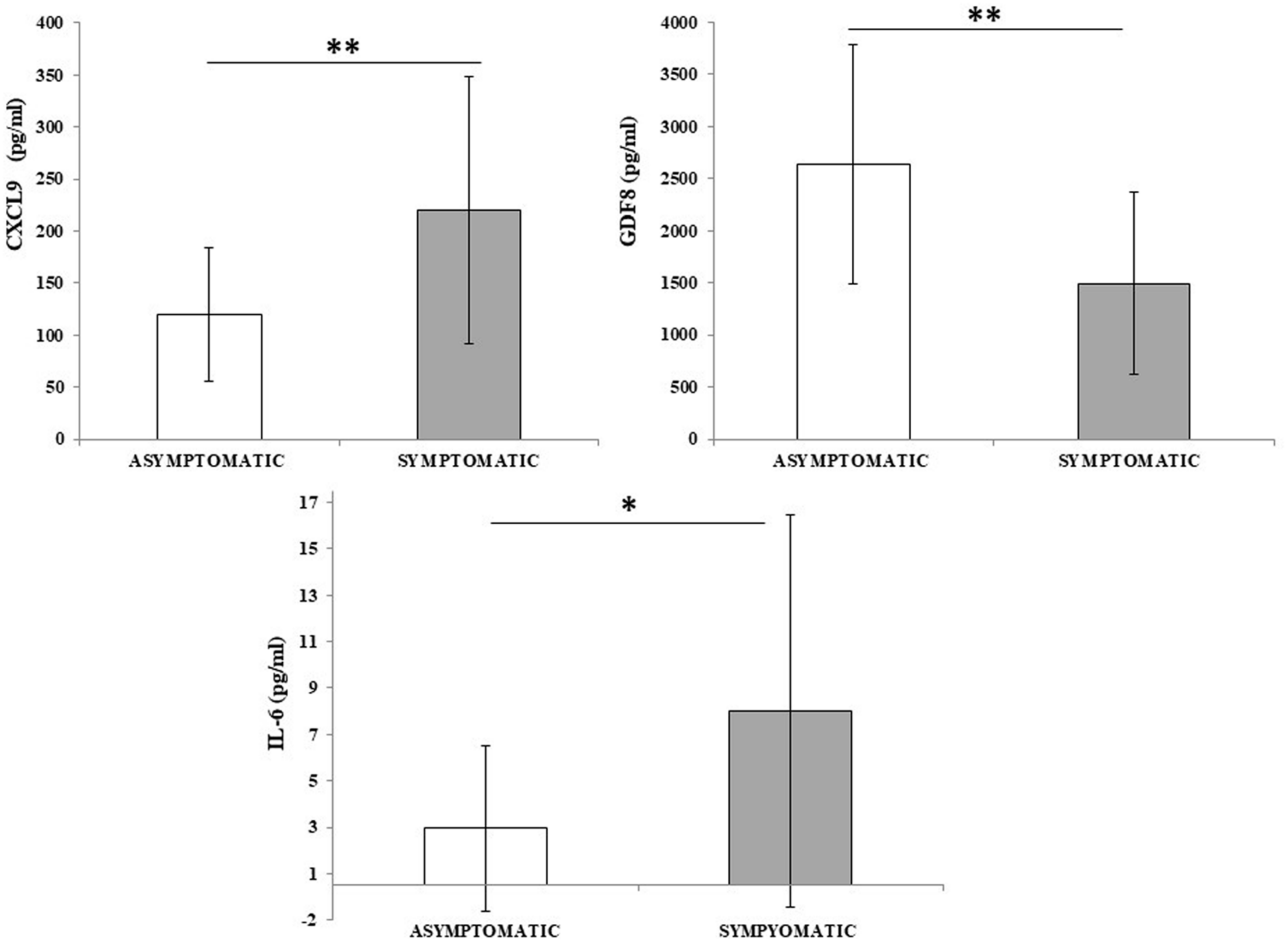
CXCL9, GDF8, and IL-6 differ between asymptomatic and symptomatic inpatients. Cytokine concentration was evaluated in plasma samples from asymptomatic inpatients (*n* = 26) and compared to symptomatic inpatients (*n* = 16). Data are reported as mean ± SD. Statistical analysis was performed with Student’s *t*-test. *p ≤ 0.05; **p ≤ 0.01.

Among all the assessed cytokines, IL-6, GDF8, and CXCL9 were statistically significant (*p* = 0.027, *p* = 0.015, and *p* = 0.0018, respectively) comparing the two groups, as reported in **Figure 2**.

**Figure 3:**
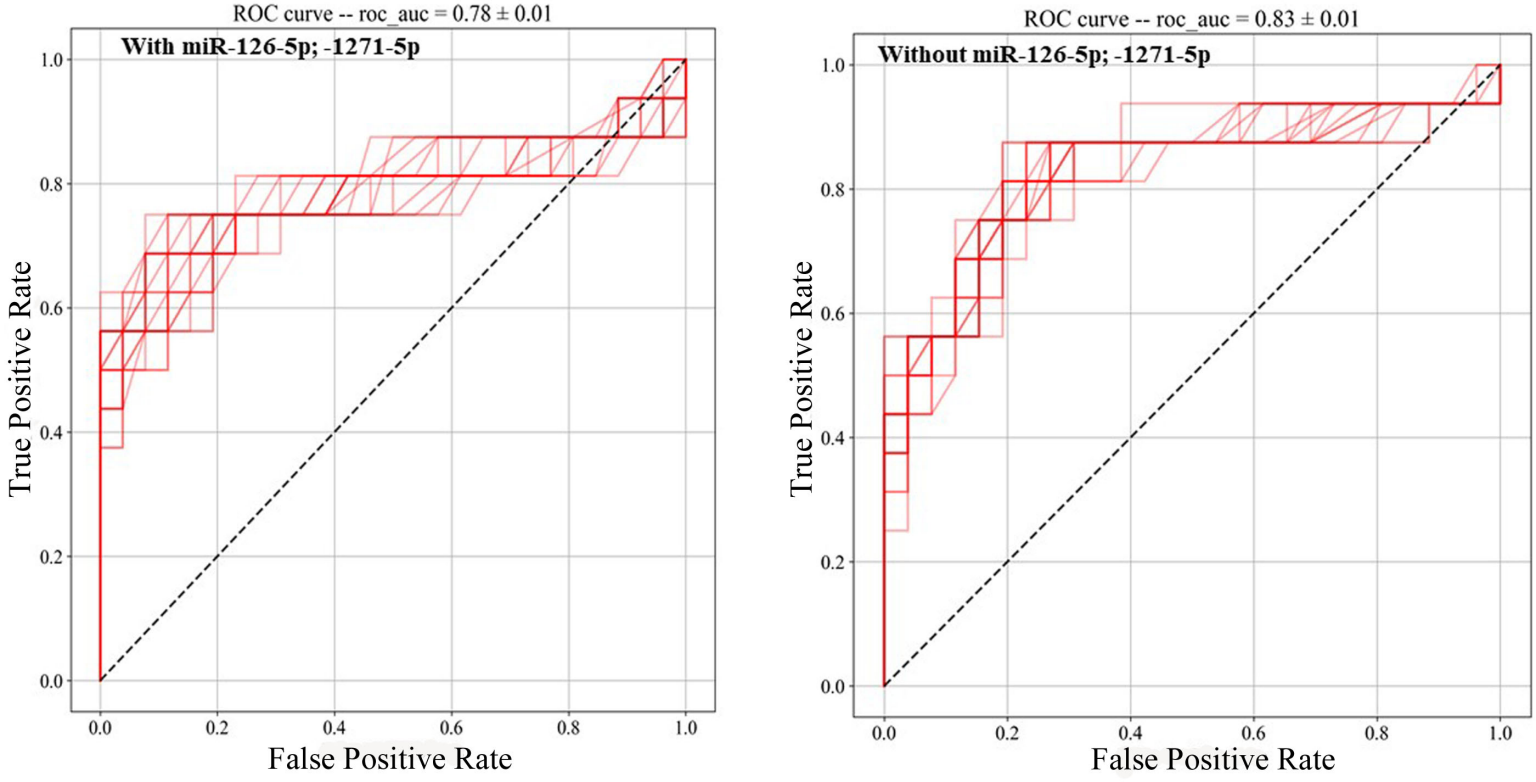
ROC analyses with the model: CXCL9, % of monocytes, albumin, CRP, with and without miR-126–5p and miR-1271–5p. ROC analyses were obtained with a machine learning-based approach (see Methods). A 10× repetition of the analyses resulted in the SD calculation and *p*-values. The model with CXCL9 and without the identified miRs resulted in the most significant one (AUC = 0.83, 95% c.i. [0.85, 0.81], *p* = 0.0028).

MiR-126–5p and miR-1271–5p ΔCt (or expression levels) combined with albumin concentration, percentage of monocytes, CRP, and the three significant cytokines were tested with the leave-one-out cross-validation approach for the best model by means of AUC-ROC values (with standard deviation and *p*-values), as reported in **Table 3**. The optimal model was determined to include CXCL9, percentage of monocyte, albumin, and CRP. Together, these variables were capable of distinguishing between symptomatic and asymptomatic inpatients (AUC = 0.83, 95% c.i. [0.85, 0.81], *p* = 0.028), as depicted in **Figure 3**, where the two AUC-ROC curves are displayed with and without the two selected miRs.

**Table 3:** AUC-ROC values with models including or excluding the selected c-miRs (miR-126–5p and miR-1271–5p).

GDF8	CXCL9	IL-6	c-miRs	AUC ± SD	*p*
X	X	X	With	0.81 ± 0.02	0.0054
			Without	0.82 ± 0.02	0.0041
X	X	–	With	0.82 ± 0.02	0.0053
			Without	0.83 ± 0.02	0.0048
–	–	X	With	0.74 ± 0.02	0.0032
			Without	0.73 ± 0.02	0.0051
X	–	–	With	0.80 ± 0.01	0.0049
			Without	0.78 ± 0.01	0.0056
–	X	–	With	0.78 ± 0.01	0.0032
			Without	**0.83 ± 0.01**	**0.0028**
–	X	X	With	0.74 ± 0.01	0.0054
			Without	0.77 ± 0.02	0.0056
X	–	X	With	0.80 ± 0.01	0.0058
			Without	0.80 ± 0.02	0.0055
–	–	–	With	0.78 ± 0.02	0.0038
			Without	0.78 ± 0.02	0.0035

The three variables, i.e., % of monocytes, albumin, and CRP concentrations, were kept in all models. The symbol X means that the specific variable has been included in the model. In bold the most significant model.

## 4 Discussion

Atherosclerosis is a chronic arterial disease that typically manifests more frequently as individuals age, although early stages can be observed in children and teenagers, highlighting the pervasive nature of artery disease development across different age groups (28, 29). This phenomenon poses a significant global health burden.

As highlighted in a recent review by our team (6), carotid plaque lesions can be viewed as tissue-specific manifestations of accelerated aging, akin to chronic pro-inflammatory macro-niches. The molecular and cellular events occurring within these niches encompass well-recognized mechanisms of the aging process, including cellular senescence characterized by a senescence-associated secretory phenotype.

Given these molecular alterations, identifying blood-circulating biomarkers predictive of carotid stenosis is a reasonable challenge. This endeavor is in line with the goals of the current Age.It project, which embraces significant translational power for preventive medicine within the CVD field. The successful identification of such biomarkers could have a substantial impact on healthcare systems, facilitating early detection and intervention strategies to mitigate the burden of CVDs. In fact, our work wants to highlight, as a pilot study, new assessable biomarkers, such as c-miRs and cytokines/chemokines, for carotid artery disease.

As a first approach, the difference between the two groups of asymptomatic and symptomatic inpatients (with carotid occlusion ≥70%) was considered to identify blood molecules shared with different concentrations, and symptomatic inpatients were regarded as those at the highest cardiovascular risk. These molecules will be further assessed as a predictive model in patients with different percentages of carotid stenoses in follow-up monitoring, as proposed in the ongoing National Italian project Age.It. Thus, the molecular signature could likely be applicable for the identification of subjects with a high probability of developing cerebrovascular events in the near future. The simultaneous detection of miRs in the two districts (plaque and blood) could give informative results on circulating miR-based signature in association with a specific plaque phenotype. However, results obtained in the discovery phase were not confirmed in the validation phase, likely due to the increase of variability among inpatients, including the increase of Ct (>32) in some samples (no detection). Similarly, our previous studies, focused on tissue versus blood miR-levels, led to comparable conclusions (25); i.e., card/arrays technology results are often not reproducible in RT-qPCR. This is an additional reason to promote the validation phase. However, the two c-miRs, miR-126–5p and miR-1271–5p, showed similar trends when comparing the two different technologies.

Thus, we identified two c-miRs, i.e., miR-126–5p and miR-1271-5p, differently expressed in plasma from asymptomatic vs. symptomatic inpatients, with both levels being higher in symptomatic individuals. Actually, the two miRs did not correlate with the plaque state (hemorrhagic vs. non-hemorrhagic), but further specific investigations on the various features and parameters of plaques in asymptomatic and symptomatic are currently underway (manuscript in preparation). These investigations are relevant due to the well-recognized role of plaque characteristics in determining cerebrovascular risk (30–33).

As far as miR-126–5p is concerned, it has received less attention than miR-126–3p even if both derive from the same pre-miRNA-126, which maps on human chromosome 9, and it is the principal miR expressed in endothelial cells. MiR-126 has a well-recognized role in the function, integrity, and proliferation of endothelial cells (34–36). In particular, recent data suggest the anti-atherosclerotic role of miR-126–5p through non-canonical, nuclear inhibition of caspase-3, thus conferring endothelial protection from stress through autophagy (37). The current work does not identify differences of miR-126–5p levels between plaques from symptomatic and asymptomatic patients, thus suggesting that higher levels of this miR could be necessary for its function. In this respect, miR-126–5p has been found to be downregulated in human atherosclerotic lesions (34), thus supporting the general hypothesis of the crucial role of the level of miR-126–5p expression. In addition, the-3p has been defined as inflamma-miR (12) and is involved in the regulation of the inflammatory pathway. Interestingly, both c-miR-126–3p and -5p have been proposed as biomarkers of cardiovascular risk in association with stable or vulnerable coronary artery plaque (38). In this perspective, the results here reported are aligned with those previously published, with c-miR-126–5p blood level being higher in symptomatic patients and assuming their highest cardiovascular risk.

As far as c-miR-1271–5p is concerned, a recent article suggests that blood c-miR-1271–5p is involved with other regulatory circular RNA in the progression of thoracic aortic dissection (39), but its role in atherosclerosis and carotid artery disease has yet to be explored.

However, the current results suggest that the two identified c-miRs are not able to fully distinguish (with sensitivity and specificity) the two groups of inpatients, and only by adding other biomarkers is it possible to obtain a molecular signature able to improve the distinction of asymptomatic and symptomatic inpatients.

The identified signature comprises three hemato-biochemical parameters i.e., the percentage of monocyte, albumin, and CRP. Indeed, the comparison of asymptomatic and symptomatic inpatients through hemato-biochemical analysis revealed striking similarities between the two groups. In particular, CRP was not statistically significant comparing directly the two groups, but the machine-learning-based approach was able to select this parameter as informative for the best model, based on the whole dataset.

Among the plethora of various cytokines/molecules assessed within the framework of the ongoing Italian National Project (PNRR-PE8: Age.It), only three of them were deemed significant and able to improve the molecular signature, thereby enhancing the distinction between asymptomatic and symptomatic inpatients. These pivotal molecules include IL-6, GDF8, and CXCL9.

These three cytokines significantly enhance sensitivity and specificity in distinguishing between the two groups. Among them, CXCL9 currently stands out as the most promising chemokine to be included in the signature, alongside the percentage of monocyte, albumin, and CRP (AUC = 0.83, *p* = 0.0028). This finding suggests the potential exclusion of the two identified miRs.

As far as the three circulating cytokines are concerned, IL-6 is a well-known pro-inflammatory cytokine at the systemic level (40); increased serum level of GDF8 has been recently associated with brachial diastolic pressure and with carotid-femoral pulse wave velocity, a measure of aortic stiffness, in healthy young male adolescents (41). Actually, a recent literature suggests a role of GDF8 in the plaque development (42), and a relationship of plasma GDF8 concentration with chronic kidney disease has been found (43–45). Our data revealed a lower plasma concentration of GDF8 in symptomatic than in asymptomatic inpatients, but activin A/follistatin, recognized to be involved together with GDF8 in the muscle metabolism, were not statistically different between the two groups. Thus, the biological meaning of these results remains to be further explored. As far as CXCL9 is concerned, it is a well-known pro-inflammatory chemokine, also termed monokine induced by gamma interferon (MIG). Interestingly, this chemokine has been associated with old phenotype/poor vascular function and multimorbidity in the so-called inflammatory aging clock (iAge), as the strongest contributor to iAge (46). In addition, the same chemokine has been proposed to be involved in plaque development and its inflammatory environment (24). In this respect, recent research supports the role of the receptor CXCR3 in plaque development. CXCR3, which binds to CXCL9, CXCL10, and CXCL11, has been shown to play a role in human smooth muscle cells in an *in vitro* model (47), highlighting the potential duality of CXCL9 as both an inflammatory plaque driver and a circulating biomarker. Various chemokines, such as CXCL10 and CCL2, have been extensively investigated for their roles in plaque development over many years, with recent studies also noting the protective role of CXCR4 (48–50). However, the current work focuses on identifying circulating biomarkers, and CXCL9 appears to be a very promising candidate for further testing.

In fact, the next challenge will be to test the identified molecular signature in patients with different carotid stenosis aiming to estimate the prediction of artery occlusion. In fact, the next stage of the current pilot study will be the possibility to assess the identified molecules in an enlarged cohort with monitored outpatients having a different level of carotid stenosis and different comorbidities in the framework of the National Plan of Resilience and Research (PNRR) i.e., Age.it.

Nevertheless, the current article exhibits some weaknesses due to the relatively small number of inpatients, as it is a pilot study and it requires replication in an independent cohort. Additionally, card/array results were not confirmed in RT-qPCR, except the trends of the two c-miRs (miR-126–5p and miR-1271–5p), which were finally validated. On the other hand, the two identified miRs do not appear to be strictly necessary for the molecular signature or for potential application as a screening test. In this regard, the use of c-miRs as biomarkers has yet to gain consensus within the scientific community and particularly concerning CVDs (51). However, other techniques are rapidly advancing to provide more reproducible measurements.

Overall, the challenge of biomarkers for the identification of biological aging (52) and predictive models of age-associated pathology development is still a growing field with expected results not only for each citizen in terms of healthy life span increase, but also for the economic impact on the healthcare system.

## Data availability statement

The data supporting the conclusions will be made available upon private request, without undue reservation.

## Ethics statement

The studies involving humans were approved by Comitato Etico Area Vasta Emilia Centro (AVEC). The studies were conducted in accordance with the local legislation and institutional requirements. The participants provided their written informed consent to participate in this study.

## Author contributions

MCa: Conceptualization, Funding acquisition, Supervision, Writing – original draft. SF: Data curation, Project administration, Writing – review & editing. SCo: Data curation, Investigation, Methodology, Writing – review & editing. EG: Data curation, Visualization, Writing – review & editing. SCa: Data curation, Investigation, Visualization, Writing – review & editing. FF: Data curation, Writing – review & editing. EC: Data curation, Supervision, Writing – review & editing. MCo: Visualization, Writing – review & editing. FO: Supervision, Writing – review & editing. IU: Data curation, Writing – review & editing. RP: Data curation, Writing – review & editing. AV: Data curation, Writing – review & editing. AA: Data curation, Writing – review & editing. FV: Data curation, Writing – review & editing. GL: Data curation, Supervision, Writing – review & editing. GP: Methodology, Supervision, Writing – original draft. MG: Methodology, Supervision, Writing – review & editing.
