# Supplementary material for: Circulating CXCL9, monocyte percentage, albumin, and C-reactive protein as a potential, non-invasive, molecular signature of carotid artery disease in 65+ patients with multimorbidity: a pilot study in Age.It
Source: Front Endocrinol (Lausanne). 2024 Jul 23;15:1407396. doi: 10.3389/fendo.2024.1407396 (PMC11300199; doi:10.3389/fendo.2024.1407396)
Supplement: Supplementary file 1 [file Table_1.docx]

**Table S1.** Fold change values of up- and down-regulated miRs identified in plasma

comparing 4 symptomatic *vs* 4 asymptomatic inpatients with card-array technology.

|  | **C-miR** | **FC** |  | **C-miR** | **FC** |
| --- | --- | --- | --- | --- | --- |
| Up-regulated | miR-409-3p | 7.1 | Up-regulated | miR-130a | 2.5 |
|  | miR-432 | 6.9 |  | miR-323-3p | 2.5 |
|  | let-7a | 6.0 |  | miR-128a | 2.5 |
|  | miR-376c | 5.6 |  | miR-24 | 2.5 |
|  | miR-134-5p | 5.2 |  | miR-301 | 2.4 |
|  | miR-376a | 4.8 |  | miR-1271-5p | 2.4 |
|  | miR-15b# | 4.6 |  | miR-26a | 2.3 |
|  | let-7e | 4.0 |  | miR-191 | 2.3 |
|  | miR-425# | 4.0 |  | miR-223# | 2.3 |
|  | miR-127 | 3.9 |  | miR-152 | 2.3 |
|  | miR-151a-5p | 3.9 |  | miR-142-5p | 2.2 |
|  | miR-133a | 3.9 |  | miR-720 | 2.2 |
|  | miR-106b# | 3.7 |  | let-7g | 2.2 |
|  | miR-145-5p | 3.5 |  | miR-223 | 2.1 |
|  | miR-361 | 3.5 |  | miR-28-3p | 2.1 |
|  | miR-769-5p | 3.5 |  | miR-126-5p | 2.1 |
|  | miR-22# | 3.5 |  | miR-143 | 2.1 |
|  | miR-15b | 3.3 |  | miR-27b | 2.1 |
|  | miR-151-3p | 3.2 |  | miR-18b | 2.1 |
|  | miR-652 | 3.1 |  | miR-181a | 2.0 |
|  | miR-221 | 3.1 |  | miR-340 | 2.0 |
|  | miR-766 | 3.0 | Down-regulated | miR-548a | -2.1 |
|  | miR-199a-3p | 3.0 |  | miR-34b | -2.2 |
|  | miR-26b# | 2.9 |  | miR-660 | -2.2 |
|  | miR-103 | 2.9 |  | miR-25 | -2.3 |
|  | miR-744 | 2.8 |  | miR-192 | -2.3 |
|  | miR-339-3p | 2.8 |  | miR-34a | -2.5 |
|  | miR-628-3p | 2.8 |  | miR-451 | -2.7 |
|  | miR-335 | 2.6 |  | miR-486 | -2.9 |
|  | miR-340# | 2.6 |  | miR-548c | -3.1 |
|  | miR-328 | 2.6 |  | miR-486-3p | -3.2 |
|  | miR-28 | 2.5 |  |  | |

**Table S2.** Fold change values of up- and down-regulated miRs identified in carotid

plaque comparing 4 symptomatic *vs.* 4 asymptomatic inpatients with card-array technology.

|  | **MiR** | **FC** |  | **MiR** | **FC** |
| --- | --- | --- | --- | --- | --- |
| Up-regulated | miR-299-5p | 4.0 | Down-regulated | miR-213 | -2.4 |
|  | miR-193b# | 3.8 |  | miR-1290 | -2.4 |
|  | miR-433 | 3.1 |  | miR-451 | -2.4 |
|  | miR-145-5p | 2.9 |  | miR-142-3p | -2.5 |
|  | miR-550 | 2.9 |  | miR-141 | -2.6 |
|  | miR-1254 | 2.6 |  | miR-1183 | -2.6 |
|  | miR-1271-5p | 2.5 |  | miR-30a-5p | -2.7 |
|  | miR-9 | 2.3 |  | miR-144# | -2.7 |
|  | miR-302b | 2.3 |  | miR-639 | -2.7 |
|  | miR-151a-5p | 2.2 |  | miR-126-5p | -2.7 |
|  | miR-454# | 2.1 |  | miR-34b | -2.7 |
|  | miR-181c# | 2.1 |  | miR-31# | -2.7 |
|  | miR-134-5p | 2.0 |  | miR-181a | -2.8 |
|  | miR-543 | 2.0 |  | miR-523 | -2.8 |
| Down-regulated | miR-148a | -2.0 |  | miR-224 | -2.8 |
|  | miR-720 | -2.0 |  | miR-1260 | -2.8 |
|  | miR-363 | -2.0 |  | miR-137 | -2.9 |
|  | miR-503 | -2.1 |  | miR-206 | -3.0 |
|  | miR-378 | -2.1 |  | miR-202 | -3.3 |
|  | miR-30d | -2.3 |  | miR-29c | -3.5 |
|  | miR-135b | -2.3 |  | miR-520c-3p | -3.9 |
|  | miR-1274A | -2.3 |  | miR-302a | -5.9 |
|  | miR-1825 | -2.4 |  |  |  |
