# Supplementary material for: Circulating CXCL9, monocyte percentage, albumin, and C-reactive protein as a potential, non-invasive, molecular signature of carotid artery disease in 65+ patients with multimorbidity: a pilot study in Age.It
Source: Front Endocrinol (Lausanne). 2024 Jul 23;15:1407396. doi: 10.3389/fendo.2024.1407396 (PMC11300199; doi:10.3389/fendo.2024.1407396)
Supplement: Supplementary file 2 [file Table_2.docx]

| miRs  (Card/array) | Plaque-A  mean ± sd | Plaque-S  mean ± sd |  | Plasma- A  mean ± sd | Plasma- S  mean ± sd |  |
| --- | --- | --- | --- | --- | --- | --- |
| miR-126-5p | 111 ± 76 | 68 ± 75 |  | 202 ± 118 | 395 ± 141 |  |
| miR-145-5p | 3343 ± 2618 | 11352 ± 13067 |  | 5 ± 2 | 19 ± 12 |  |
| miR-151-5p | 44 ± 21 | 93 ± 53 |  | 39 ± 16 | 158 ± 80 |  |
| miR-451a | 201 ± 177 | 68 ± 33 |  | 1751 ± 869 | 674 ± 324 |  |
| miR-720 | 52320 ± 26015 | 32340 ± 20148 |  | 54 ± 14 | 121 ± 35 |  |
| miR-1271-5p | 4 ± 2 | 11 ± 6 |  | 2 ± 2 | 5 ± 3 |  |
| miR-134 | 0.4 ± 0.2 | 1.0 ± 0.9 |  | 0.37 ± 0.45 | 6 ± 7 |  |
| miR-34b | 4 ± 1 | 2 ± 1 |  | 38 ± 21 | 30 ± 32 |  |

Table S3. Relative expression of the selected miRs obtained by card/array technology both in plaque biopsies and plasma from the same 8 inpatients.

A = Asymptomatic (n 4); S = Symptomatic (n 4)
